# A dual inhibitor of the proteasome catalytic subunits LMP2 and Y attenuates disease progression in mouse models of Alzheimer’s disease

**DOI:** 10.1038/s41598-019-54846-z

**Published:** 2019-12-05

**Authors:** In Jun Yeo, Min Jae Lee, Ahruem Baek, Zachary Miller, Deepak Bhattarai, Yu Mi Baek, Hyun Jung Jeong, Yun Kyung Kim, Dong-Eun Kim, Jin Tae Hong, Kyung Bo Kim

**Affiliations:** 1 College of Pharmacy, Chungbuk National University, Cheongju, Chungbuk 28160 Republic of Korea; 2 Department of Pharmaceutical Sciences, University of Kentucky, Lexington, KY 40536-0596 USA; 3 Department of Bioscience and Biotechnology, Konkuk University, Seoul, 05029 Republic of Korea; 4 Korea Institute of Science and Technology (KIST), Brain Science Institute, Convergence Research Center for Diagnosis, Treatment and Care System of Dementia, Seoul, 02792 Republic of Korea

**Keywords:** Chemical biology, Drug discovery

## Abstract

The immunoproteasome (iP) is a variant of the constitutive proteasome (cP) that is abundantly expressed in immune cells which can also be induced in somatic cells by cytokines such as TNF-α or IFN-γ. Accumulating evidence support that the iP is closely linked to multiple facets of inflammatory response, eventually leading to the development of several iP inhibitors as potential therapeutic agents for autoimmune diseases. Recent studies also found that the iP is upregulated in reactive glial cells surrounding amyloid β (Aβ) deposits in brains of Alzheimer’s disease (AD) patients, but the role it plays in the pathogenesis of AD remains unclear. In this study, we investigated the effects of several proteasome inhibitors on cognitive function in AD mouse models and found that YU102, a dual inhibitor of the iP catalytic subunit LMP2 and the cP catalytic subunit Y, ameliorates cognitive impairments in AD mouse models without affecting Aβ deposition. The data obtained from our investigation revealed that YU102 suppresses the secretion of inflammatory cytokines from microglial cells. Overall, this study indicates that there may exist a potential link between LMP2/Y and microglia-mediated neuroinflammation and that inhibition of these subunits may offer a new therapeutic strategy for AD.

## Introduction

Alzheimer’s disease (AD) is the most common form of dementia in the elderly and presents one of the greatest health-care challenges further exacerbated by an aging global population. Over the past decades, extracellular β-amyloid peptide (Aβ) plaques and intraneuronal neurofibrillary tangles (NFT) composed of hyperphosphorylated tau protein have been established as major hallmarks of AD^1^. As a result, a major effort has been put forth in developing drugs that can reduce neurofibrillary tangles and amyloid plaques, long suspected to be a major cause of AD (‘the amyloid hypothesis’). However, none of drugs targeting Aβ formation or clearance have succeeded in clinical trials thus far^2,3^, questioning the validity of the long-held prevailing hypothesis. In addition, while therapies which target tau aggregation are in active development, as yet no effective therapies are available^4,5^.

In mammalian, most intracellular proteins, ranging from defective ribosomal products (DRiPs) to signaling proteins regulating numerous cellular processes (e.g., cell cycle control, immune response, apoptosis, stress response), are destined for degradation by an ATP- and ubiquitin-dependent process that involves the proteasome, an evolutionarily-conserved multiprotease complex^6^. The proteasome 20S core is composed of four stacked heptameric rings: two outer α-rings and two inner β-rings. Each β-ring of the constitutive proteasome (cP) harbors a set of three catalytic β-subunits (X/β5, Y/β1, Z/β2) which are constitutively expressed throughout the body and display different substrate preferences, often referred to as chymotrypsin-like (CT-L), caspase-like (C-L) and trypsin-like (T-L) activities, respectively. In response to cellular stress or pro-inflammatory cytokines such as TNF-α or interferon (IFN)-γ, cells upregulate three variant forms of the proteasome catalytic subunits, known as immuno-subunits, leading to the formation of the immunoproteasome (iP)^7,8^. The iP utilizes the catalytic immuno-subunits LMP2/β1i, MECL-1/β2i and LMP7/β5i in place of the constitutive proteasome (cP) counterparts Y/β1, Z/β2 and X/β5, respectively. The iP plays an important role in MHC (major histocompatibility complex) class I antigen presentation^9^ and in the management of oxidative stress via the degradation of oxidant-damaged proteins^10,11^. In addition, the iP is shown to contribute to or modulate inflammatory responses^12–14^. Specifically, studies demonstrate that inhibition of LMP7 activity leads to suppression of pro-inflammatory cytokine release in human tissues such as T-cells, B-cells, neutrophils, or monocytes^14,15^. Eventually, this function led to the development of several LMP7 inhibitors as potential therapeutic agents for inflammatory diseases. For example, KZR-616, an epoxyketone peptide developed by Kezar Life Sciences, is currently in early clinical development for the treatment of rheumatic diseases, such as lupus nephritis (LN)^15^. Although recent studies demonstrate that co-inhibition of LMP7 and LMP2 is more effective than LMP7 inhibition alone in models of experimental autoimmune diseases^16^, it remains to be verified whether or not the other iP subunits (LMP2 and MECL-1) are involved in inflammatory responses.

There is increasing evidence that inflammation plays a key role in the pathogenesis of AD^17^. As such, several FDA-approved anti-inflammatory drugs targeting cyclooxygenases (COXs) or TNF-α have been investigated for their efficacy on AD via population-based studies^18–21^. While positive results from initial studies have spurred interest in inflammation as a target in AD, further clinical trials have yielded no effective therapies so far^22–25^. Several anti-inflammatory small molecules have also been identified in high-throughput screening campaigns, but they have yet to translate into effective AD drugs^26,27^. A recent study has shown that LMP7 gene knockout in APP/PS1 mice dramatically alters the profiles of cytokine release from microglia and improves Aβ-associated cognitive deficits^28^. Studies also found that LMP2 and LMP7 are upregulated at both the mRNA and protein levels in plaque-associated microglia and astrocytes from AD mice^29–32^. These findings have been confirmed in post-mortem human AD brains where levels of active iP but not cP subunits are increased relative to control samples^30,33^, suggesting a potential link between the iP and AD pathogenesis. However, the effect of pharmacological inhibition of iP on the pathogenesis of AD has not been investigated thus far.

Here, we report that YU102, an epoxyketone peptide-based dual inhibitor of LMP2 and Y subunits, improves cognitive function in AD mouse models without affecting Aβ deposition. In addition, we show that YU102 suppresses the secretion of inflammatory cytokines by microglial cells. Taken together, our current results suggest that the proteasome catalytic subunits LMP2 and Y are closely linked to microglia-mediated inflammatory responses and that LMP2/Y inhibition may offer a new therapeutic strategy for neuroinflammatory diseases including AD.

## Results

### YU102 improves cognitive function in an LPS-induced mouse model of neuroinflammation

**Figure 1:**
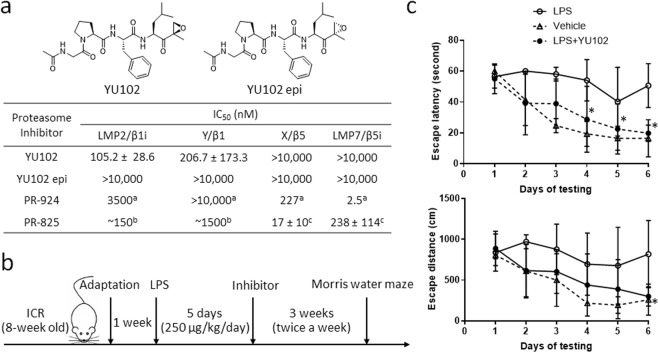
YU102 improves cognitive impairment in a mouse model of inflammation induced by LPS (lipopolysaccharide). (**a**) Proteasome inhibitory activity of immunoproteasome (iP) inhibitors (YU102, PR-924), YU102 epimer (an inactive stereoisomer of YU102) and constitutive proteasome (cP) inhibitor (PR-825). Data is shown as mean ± SD derived from a non-linear regression based on n = 3 replicates per compound per concentration. ^a^IC_50_ values were determined from competition assays in Raji cell lysates^85^. ^b^IC_50_ values were approximated from ProCISE assay using A20 murine lymphoma cells^14^. ^c^IC50 values were obtained from ProCISE ELISA using MOLT-4 human leukemia cells^86^. (**b**) A schematic depicting the experimental schedule for ICR mice. Dose of YU102 (10 mg/kg) was determined based on its *in vitro* potency relative to the *in vitro* potencies of carfilzomib and bortezomib for which effective doses were previously reported^87^. (**c**) Spatial recognition memory was evaluated by the Morris water maze test: escape latency time in the target quadrant (above) and escape distance of the mice (below). Statistical analyses of escape latency and escape distance were performed via two-way ANOVA. *Differences in escape latency on days 4–6 and distance on day 6 between LPS-treated and YU102 treated were statistically significant (p-value < 0.05, n = 5).

To determine the effects of proteasome inhibitors (PIs) including iP-selective inhibitors on cognitive function, we chose to use a lipopolysaccharide (LPS)-induced mouse model of neuroinflammation, known to display AD-like cognitive impairment. Specifically, ICR mice (8-week old) were treated with daily intraperitoneal (i.p.) injections of LPS for 5 days, followed by treatment with PR-924 (LMP7-selective)^34^, PR-825 (X-selective)^35^, YU102 (a dual inhibitor of LMP2 and Y)^36^, or conventional PIs (carfilzomib & bortezomib: targeting both X and LMP7) twice a week for 3 weeks (Fig. 1a,b). At the end of the treatment period, the Morris water maze test, a widely accepted method for examining spatial learning and memory^37^, was performed. Of note, we did not observe any irregularity in the motor activity of mice treated with vehicle, LPS or YU102. However, nearly all the mice treated with conventional PIs did not survive to complete the test, indicating the severe toxicity of these PIs in this mouse model. In contrast, the YU102-treated group displayed no overt signs of toxicity, but a significant improvement in distance and escape latency compared to the mice treated with LPS alone (Fig. 1c). Mice treated with PR-825 or PR-924 displayed only mild improvement in performance relative to LPS-treated control mice (Suppl. Fig. 1). This result is interesting in that LMP2/Y inhibitors or genetic knockout previously had no impact on inflammatory responses in human peripheral blood mononuclear cells or mouse peritoneal macrophages^38,39^. Overall, this suggests that LMP2 and/or Y function may be associated with cognitive impairment in the mouse model of neuroinflammation.

### YU102 ameliorates AD-related cognitive impairment in the Tg2576 mouse model

**Figure 2:**
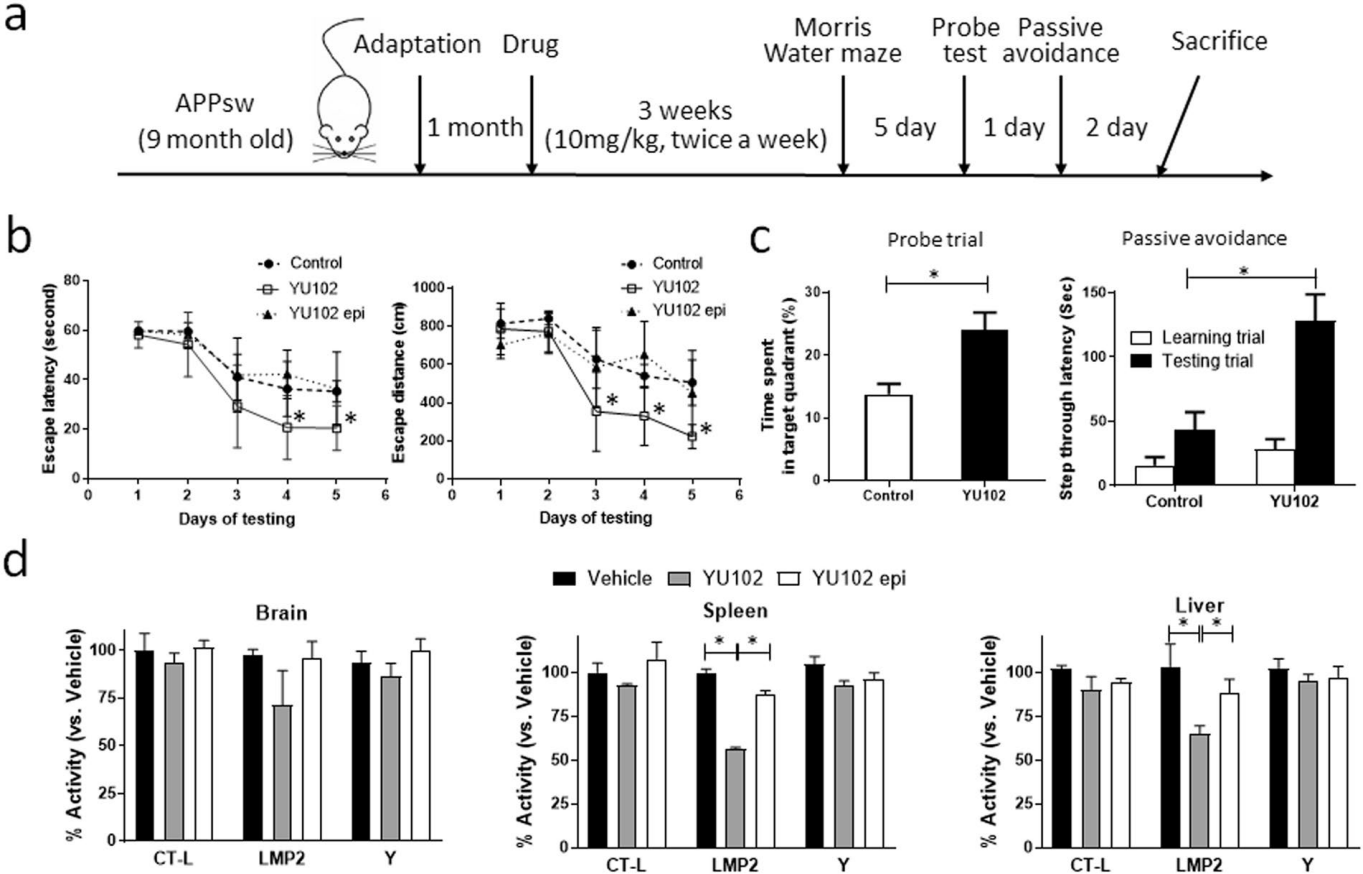
Inhibition of LMP2 improves cognitive impairment in a APP transgenic mouse model of AD. (**a**) A schematic depicting the experimental schedule for the behavior test. (**b**) YU102 ameliorates cognitive deficits in Tg2576 mice. Cognitive function in Tg2576 mice was evaluated by the Morris water maze test: escape latency time (left) and escape distance of the mice (right). Statistical analyses of escape latency and escape distance were performed via two-way ANOVA. *Difference in escape latency on days 4–5 or distance on days 3–5 between control and YU102-treated mice was statistically significant (p-value < 0.05, n = 8). (**c**) Upon the completion of the Morris water maze test, Tg2576 mice were evaluated in the probe trial (left) and passive avoidance test (right). Statistical analyses of probe trial and passive avoidance were performed via Student’s t-test. Differences in time spent in target quadrant or step through latency between control and YU102-treated mice were statistically significant (p-value < 0.05, n = 8). (**d**) Systemic and selective inhibition of LMP2 in Tg2576 by YU102. Proteasome activities in major organs (tissues) collected from mice treated with vehicle, YU102 (10 mg/kg), or YU102 epimer (10 mg/kg) were measured using fluorogenic substrates. Error bars in Fig. 2D are standard deviation derived from three technical replicates. *Differences in LMP2 inhibitory activity in spleen and liver tissues between control and YU102-treated group or YU102-treated and YU102 epi-treated group were statistically significant (p-value < 0.05, n = 3).

Next, we further verified the efficacy of YU102 in the transgenic mouse model Tg2576, which expresses human amyloid precursor protein (APP) with the Swedish double mutation (KM670/671NL) and develops age-related Aβ deposits that lead to deficits in learning and memory^40^. In this study, an inactive stereoisomer of YU102 (YU102 epi) was used as a negative control (Fig. 1a). Tg2576 mice (10-month old) were treated with YU102 or YU102 epi via i.p. injection (10 mg/kg) twice weekly for 3 weeks (Fig. 2a). Mice were then tested in the Morris water maze for 5 trial days, followed by a single probe trial on day 6 and passive avoidance test on days 7 and 8. Consistent with the results obtained from the LPS-induced inflammation model, Tg2576 mice treated with YU102 performed significantly better in terms of escape latency and distance than those treated with YU102 epi or vehicle (Fig. 2b). In the probe trial, the percentage of time spent in the target quadrant was greater for YU102-treated mice (~24%) than for vehicle-treated mice (~14%) (left, Fig. 2c), suggesting that YU102 ameliorates memory impairment in Tg2576 mice. The results from the passive avoidance test showed an average step-through latency of 128 sec for YU102-treated Tg2576 mice compared to the vehicle-treated mice with ~44 sec, further supporting the positive impact of YU102 on short-term memory impairment in Tg2576 mice (right, Fig. 2c).

At the end of behavioral testing, the Tg2576 mice were euthanized and proteasome activities in various organs were measured to examine target engagement in mice. The assessment of target engagement took advantage of the irreversible, covalent binding of YU102 to the catalytic Thr1 of proteasome catalytic subunits. Much to our surprise, the LMP2/Y dual inhibitor YU102 affected the activity of LMP2 only, but not those of Y subunit or LMP7/X subunits (referred to as the CT-L activity) (Fig. 2d, for additional organs, see Suppl. Fig. 2). Overall, weaker LMP2 inhibition was observed in the brain than in other organs. This may be due to poor brain permeability of YU102. When we investigated this possibility using a cell line overexpressing ABCB1 (RPMI 8226/ABCB1), we found that YU102 is a mild substrate of ABCB1 (Suppl. Fig. 3). Given this, we suspect that repeated injection and irreversible effect of YU102 likely contribute to overcoming low BBB permeability. Alternatively, there is a possibility that the turnover rate of proteasomes in the brain is faster than those in other organs during the 8-day interval between the final YU102 treatment and sacrifice. In addition, given unusually high specificity of the family of peptide α,β-epoxyketones including YU102 for the proteasome^41–43^, we postulate that the effect of YU102 on cognitive function is unlikely due to off-target interactions. Taken together, our data showing systemic inhibition of LMP2 but not Y in organs throughout the mice treated with YU102 (including brain) support an important contribution of LMP2 to cognitive impairment in Tg2576 mice, indicating that LMP2 inhibition alone may be sufficient to improve cognitive function in Tg2576 mice.

### YU102 exerts its efficacy independently of Aβ deposition

**Figure 3:**
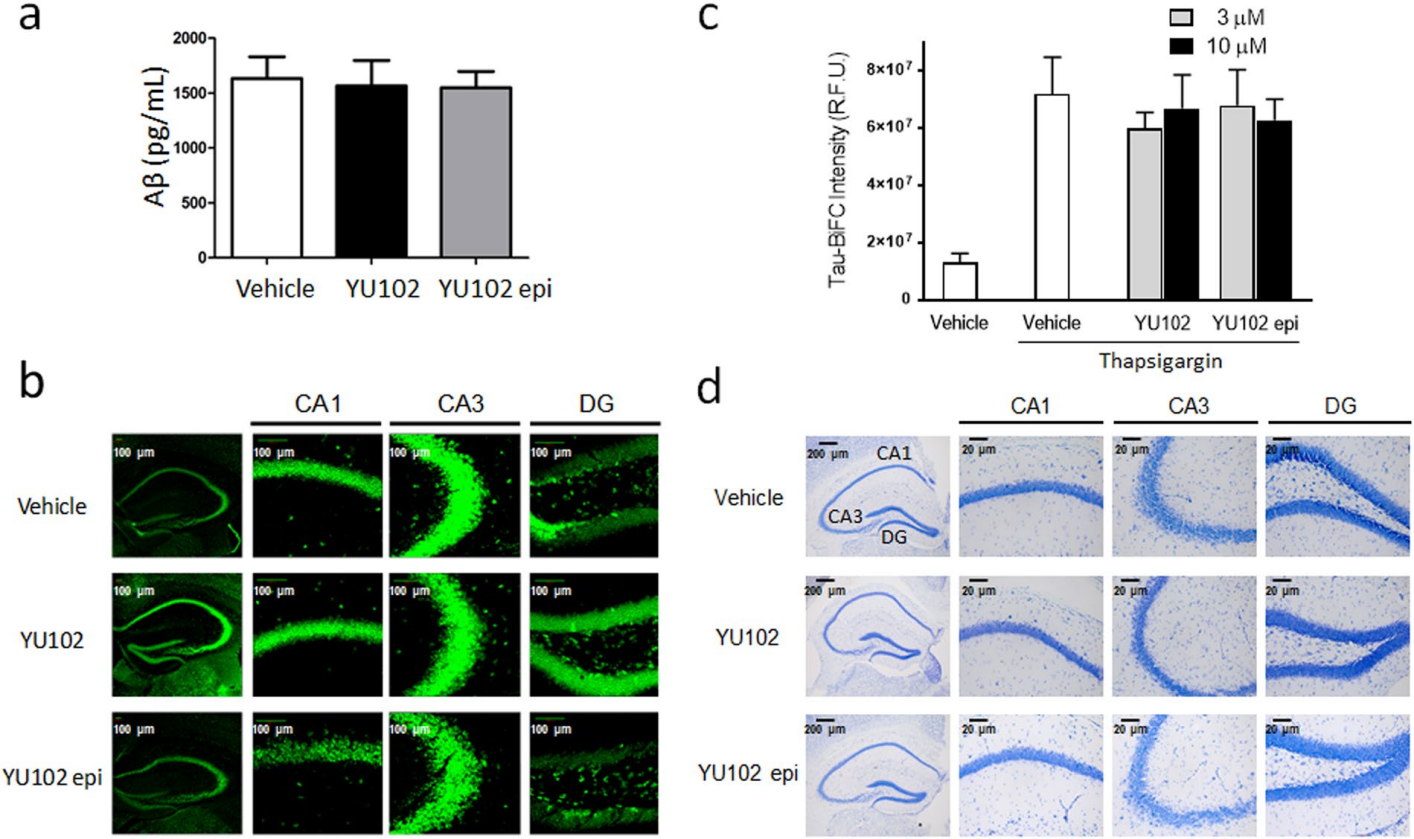
Efficacy of YU102 in Tg2576 mice is independent of Aβ deposition and tau aggregation. (**a**) ELISA-based quantification of Aβ_1–42_ in hippocampal tissues isolated from Tg2576 mice. The difference in the levels of Aβ_1–42_ between vehicle control and YU102-treated mice was not statistically significant (p-value > 0.1, n = 3). Statistical analysis of ELISA results was performed via Student’s t-test. (**b**) Thioflavin T staining of Aβ fibrils in hippocampal tissue sections from Tg2576 mice. (**c**) YU102 has no effect on tau aggregation. Thapsigargin induces tau aggregation in HEK 293 tau-BiFC cells, activating a tau BiFC fluorescence signal that can be detected. (**d**) YU102 displays no neuroprotective effects during the experimental period. Hippocampal tissues isolated from the brains of Tg2576 mice were stained with Cresyl violet, a marker for Nissl substance in neurons.

Given the presumed role of Aβ in the cognitive dysfunction of Tg2576 mice, we initially suspected that YU102 may lower Aβ deposits in the brain of Tg2576 mice (either by increasing clearance of Aβ deposits or decreasing their formation). To determine this, we measured the levels of soluble Aβ in hippocampal tissues of Tg2576 mice using an enzyme-linked immunosorbent assay (ELISA) and the levels of amyloid fibrils using the fluorescent dye Thioflavin T. The levels of soluble Aβ and Aβ fibrils did not differ between the mice treated with YU102 and vehicle-treated mice (Fig. 3a,b). The results can be cautiously interpreted that YU102 may improve cognitive function in the Tg2576 model independently of Aβ deposition or clearance. This result is potentially significant in designing non-Aβ targeting AD drugs since several drugs with proven Aβ-clearing ability have failed to demonstrate clinically meaningful efficacy in a string of recent high-profile phase 3 clinical trials^44–46^.

Tau aggregation has been investigated as a key factor in AD pathology and a potential target for therapeutic intervention^47^. In Tg2576 mice, tau undergoes hyperphosphorylation and subsequently oligomerizes in an age-dependent manner that coincides with the appearance of Aβ oligomers and declining cognitive function^48–50^. Therefore, to determine whether YU102 can inhibit the oligomerization of hyperphosphorylated tau, we employed a HEK 293 tau BiFC (bimolecular fluorescence complementation) cell-based assay^51^. In this assay, oligomerization and aggregation of hyperphosphorylated tau can be initiated by an inducer of endoplasmic reticulum (ER) stress such as thapsigargin and detected via the reconstitution of the fluorescent protein Venus. As shown in Fig. 3c, YU102 did not block thapsigargin-induced tau aggregation. Given this result, we hypothesize that it is highly unlikely that YU102 affects Aβ deposition or tau polymerization. We next examined whether treatment with YU102 has any impact on neuronal cells in Tg2576 mice by staining isolated hippocampal with Cresyl violet. The staining result showed that there is no noticeable difference in the total number of neurons between mouse groups treated with YU102, YU102 epi, or vehicle only (Fig. 3d), suggesting that YU102 had no adverse effects on neuron. In this regard, it should be noted that modeling of neuron loss has not been successful in most of currently available transgenic mouse models of AD, whereas extensive amyloid plaque pathology, inflammatory changes and behavioral deficits can be readily characterized^52,53^.

### YU102 reduces the number of reactive astrocytes and microglia in Tg2576 mice

**Figure 4:**
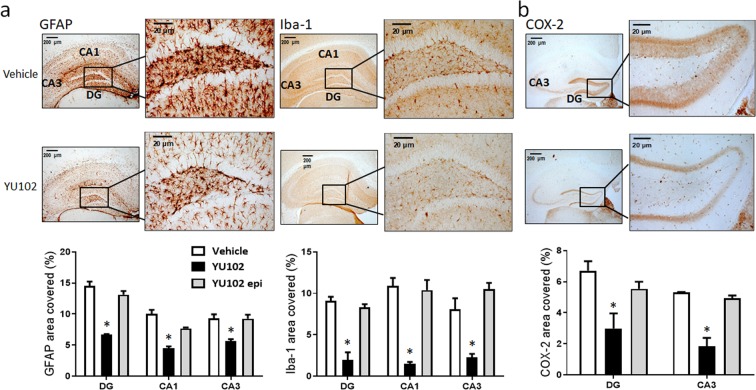
YU102 reduces the numbers of activated astrocytes and microglia. (**a**) Reactive astrocytes (left) and microglial cells (right) were visualized using their respective markers (GFAP and Iba-1) in hippocampal tissues from Tg2576 mice. (**b**) Expression levels of COX-2 in hippocampal tissues in Tg2576 mice treated with YU102 are lower than in the control Tg2576 mice. Quantifications of the areas positively labeled with GFAP, Iba-1 or COX-2 were performed using the ImageJ v1.52a downloaded from the NIH website (http://rsb.info.nih.gov/ij). Quantification results (n = 3 mice per group) were analyzed using two-way ANOVA, followed by Bonferroni posttests. Representative images of immunohistochemistry staining with respective antibodies are shown here. Full immunohistochemistry results are included in Suppl. Fig. 4.

Reactive astrocytes and microglia cells are known for their critical contributions to inflammation^17,54^ and have received considerable attention in drug discovery efforts for neurodegenerative diseases^55–57^. In *in vitro* and *in vivo* studies, Aβ and LPS have been shown to distinctly alter cytokine production profiles and induce innate immune signaling and microglial activation^58–61^. Therefore, we investigated whether YU102 has any impact on the activation of glial cells in brain tissues of Tg2576 mice. Analysis of GFAP and Iba-1 immunostaining (well-known markers of reactive astrocytes and microglia, respectively) indicated that the numbers of positively stained cells in hippocampal tissues are fewer in mice treated with YU102 than in the control group (Fig. 4a, and Suppl. Fig. 4a,b for full analysis). Expression of COX-2, a proinflammatory enzyme upregulated in human AD and mouse AD models^62^, was also reduced in hippocampal tissues of mice treated with YU102 (Fig. 4b, and Suppl. Fig. 4c for full analysis). These data collectively suggest that LMP2/Y is involved in the activation of glia cells and that LMP2/Y inhibition suppresses the activation of astrocytes and microglia and associated inflammatory responses.

### YU102 attenuates the secretion of pro-inflammatory cytokines from microglia

**Figure 5:**
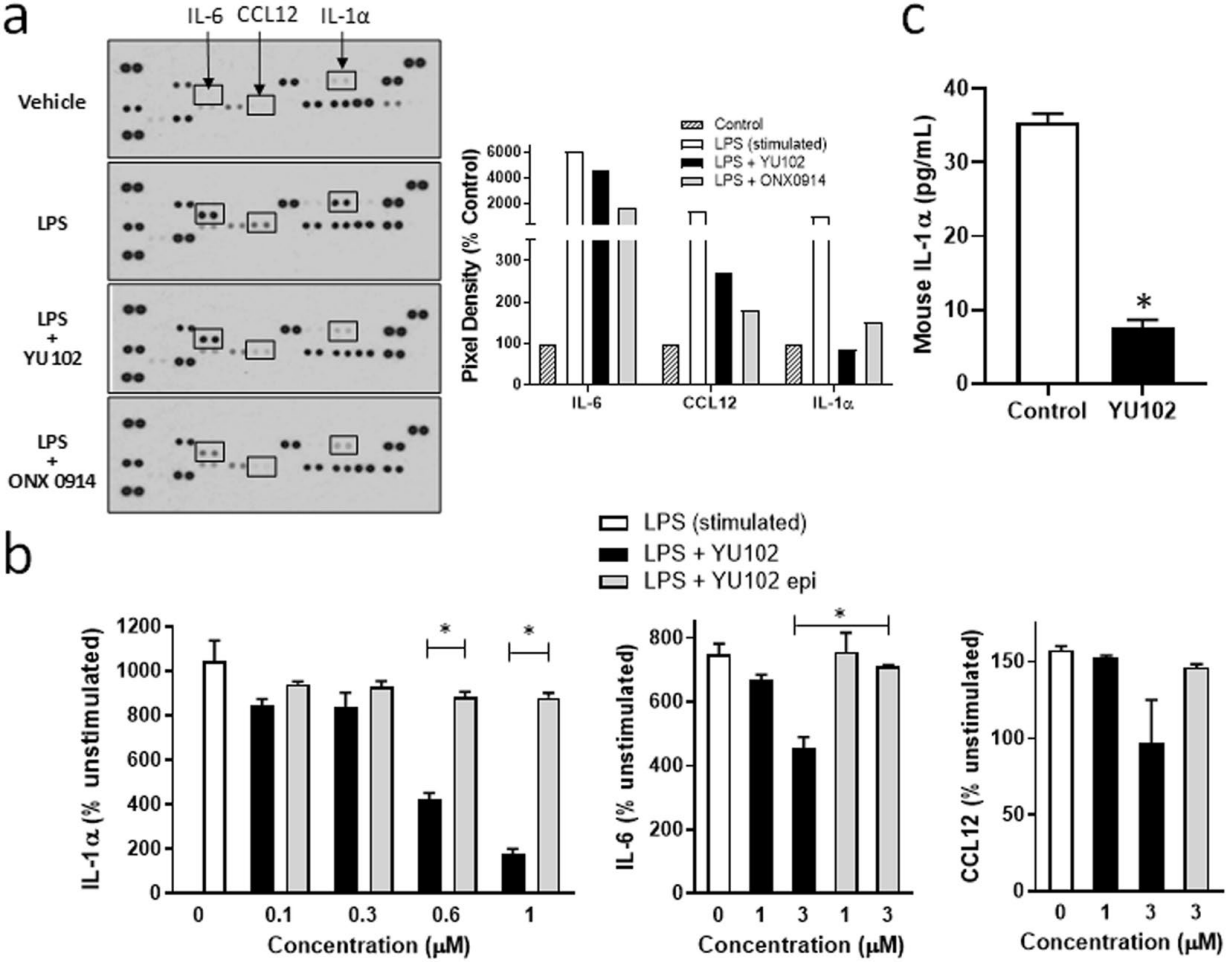
Suppression of cytokine production by YU102 in LPS-stimulated BV-2 cells. (**a**) Cytokine and chemokine protein array blots of BV-2 cells treated with vehicle, LPS (1 μg/mL) alone, and YU102 (3 μM) or ONX0914 (3 μM) with LPS (1 μg/mL). Arrow labels indicate cytokines that are most significantly impacted by YU102. Graph depicts the fold change of each cytokine or chemokine (mean). The signal intensity of each cytokine or chemokine was expressed relative to the mean of the intensity of the corresponding spots from vehicle control sample. (**b**) Cytokine production in LPS-stimulated BV-2 cells with and without YU102 was determined by ELISA. Supernatants of BV-2 cells from 12-well culture plates were used to measure quantities of released cytokines. BV-2 cells were incubated with LPS (1 μg/mL) and YU102 or YU102 epimer for 24 h. All values are expressed as mean ± SEM from three independent experiments. *Differences in suppression of IL-1α and IL-6 levels between YU102-treated and YU102 epi-treated group were statistically significant (p-value < 0.05, n = 3). (**c**) ELISA-based quantification of serum IL-1α levels (5-fold diluted) in Tg2576 mice. Serum IL-1α levels were significantly lower in YU102-treated Tg2576 mice than in vehicle controls (p-value < 0.05, n = 3). Statistical analysis of ELISA results was performed via Student’s t-test.

Production of pro-inflammatory cytokines by microglial cells and associated changes in phagocytic properties are considered to be major contributing factors to the recently recognized “cellular” phase of Alzheimer’s disease^63,64^. We thus examined whether YU102 can suppress the secretion of inflammatory cytokines by microglial cells using an immortalized murine microglial cell line BV-2 (commonly used as a substitute for primary microglia in many experimental settings)^65^. BV-2 cells were incubated with LPS (1 µg/mL) with and without YU102 (3 µM), following a procedure previously reported^66–68^. The LMP7-selective inhibitor ONX 0914 was used as a positive control^14^. After an additional 24 h incubation, culture media was collected and analyzed for the levels of 40 cytokines and chemokines using a membrane antibody array. BV-2 cells treated with LPS only exhibited elevated levels of multiple pro-inflammatory cytokines compared to unstimulated cells (Fig. 5a) (for full results, see Suppl. Fig. 5). As previously reported^14^, ONX 0914 suppressed the LPS-triggered secretion of pro-inflammatory cytokines, such as IL-1α, IL-1β, CCL12/MCP-5, IL-6 and CCL5/RANTES. YU102 also inhibited cytokine release but displayed a slightly different inhibition profile, suppressing production of IL-1α, IL-1β, CCL12/MCP-5 and to a lesser degree, IL-6. These results were further corroborated by the ELISA result showing the reduced production of IL-1α, IL-6, and CCL12 in LPS-stimulated BV-2 cells, with IL-1α being most sensitive to drug treatment (Fig. 5b).

Given the result showing a substantial decrease in the levels of IL-1α in YU102-treated inflammatory BV-2 cells compared to vehicle controls, we next examined whether the levels of IL-1α in Tg2576 mice are also affected by YU102 treatment. As shown in Fig. 5c, YU102 treatment led to significantly decreased serum levels of IL-1α in Tg2576 mice compared to vehicle controls, suggesting that YU102 may contribute to suppressing inflammatory responses in the mouse model of AD. While the detailed understanding on the specific role of IL-1α in disease progression needs to be further dissected, it has been recently reported that IL-1α secreted by activated microglia contributes to the induction of a subtype of reactive astrocytes, termed A1, which induce the death of neurons and oligodendrocytes and consequently lead to cognitive deficits^69^. Given this, the result obtained by us suggest that LMP2/Y may be linked to microglia-mediated neuroinflammation pathway via modulating cytokine release.

### Impacts of YU102 on degeneration of retinal pigment epithelium in Tg2576 mice

**Figure 6:**
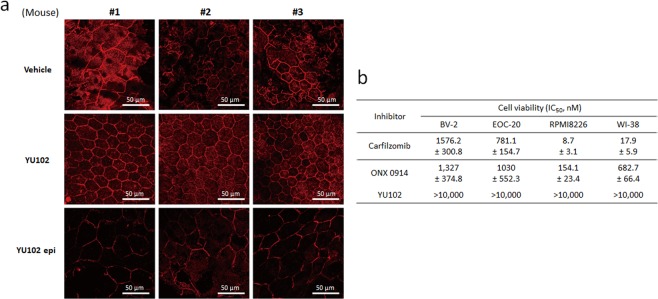
YU102 inhibits *in vivo* and *in vitro* RPE (retinal pigment epithelium) degeneration. (**a**) RPE from eyes of Tg2576 mice treated with vehicle, YU102 or YU102 epi were isolated and immunostained to establish boundaries of RPE monolayers. (**b**) Cell viability for carfilzomib, ONX0914 or YU102 in various cell lines.

Inflammation triggered by Aβ is widely considered to be a major contributor to retinal pigment epithelium (RPE) abnormalities in APP transgenic animal models including Tg2576^70,71^. Therefore, we examined whether YU102 affects the structural integrity of RPE in Tg2576 mice via suppressing cytokine release from microglia. RPE samples were collected from Tg2576 mice treated with vehicle, YU102, or YU102 epi. When immunohistochemical staining was performed with an anti-β-catenin primary antibody followed by Alexa 555-conjugated secondary antibody, the orderly mosaic structure of RPE typically observed in non-transgenic mice was severely damaged in Tg2576 mice treated with vehicle only (top, Fig. 6a), as previously reported^72^. However, YU102 provided Tg2576 mice with almost complete protection from RPE damage, showing the typical mosaic structure of RPE (middle, Fig. 6a). In contrast, YU102 epi (an inactive stereoisomer of YU102) provided no protection from RPE mosaic disruption (bottom, Fig. 6a). Taken together, this result further supports that LMP2/Y function is linked to inflammatory responses triggered by Aβ in Tg2576 mice.

### YU102 has no cytotoxic effect

Throughout our *in vivo* efficacy studies, we observed no overt signs of toxicity in mice treated with YU102. To further investigate this, we incubated a panel of cell lines (two murine microglial cell lines, BV-2 and EOC-20; a human myeloma cell line, RPMI 8266; a human lung fibroblast cell line, WI-38) with YU102, ONX 0914 (LMP7-selective), or carfilzomib (an FDA-approved inhibitor targeting multiple proteasome subunits including X and LMP7, a positive control known to induce cell death) and performed cell viability assays. YU102 showed no negative effects on the viability of all four cell lines at the pharmacologically relevant concentrations (Fig. 6b). In comparison, ONX 0914 was much more cytotoxic than YU102. This result is consistent with previous studies showing near complete cell death in a primary neuron model after 48 hours treatment with 500 nM ONX 0914^73^. In addition, we found that LPS consistently sensitizes EOC-20 microglial cells to ONX 0914 but not to YU102 (Suppl. Fig. 6).

## Discussion

An estimated 5.8 million Americans of all ages are living with AD in 2019 and this number is projected to rise to 14 million people by 2050 with a projected cost of more than $1.1 trillion (https://www.alz.org). Despite this looming health and economic crisis, only symptomatic treatments are currently available. To address this issue, a great amount of effort has been put forth to develop disease-modifying drugs without much success to date.

In the current study, we showed that the LMP2/Y dual inhibitor YU102 ameliorates cognitive deficits in mouse models of AD, independent of Aβ deposition or tau aggregation. Cytokine membrane array data and decreased numbers of activated glia *in vivo* indicate that YU102 may improve cognitive function via suppressing inflammatory cytokine release by microglial cells. Among the cytokines examined, the secretion of IL-1α was most affected by YU102 in LPS-stimulated microglial cells, consistent with the previously reported role of IL-1α in microglia-mediated neuroinflammation^69^. While we were unable to assess the levels of IL-1α in the brain of Tg2576 mice through ELISA to provide meaningful interpretations, it was noted that serum IL-1α levels were significantly lower in YU102-treated Tg2576 mice than in vehicle controls. Combined with the previous reports demonstrating that inflammatory cytokines secreted by activated microglia induce a subtype of reactive astrocytes, termed A1, which induces the death of neurons and oligodendrocytes^69^, our data collectively suggest that YU102 exerts its efficacy via suppression of IL-1α-mediated microglial inflammatory responses. While a relatively minor contribution of peripheral immune cells (macrophages) in AD progression has been also suggested^74^, the impact of YU102 on these cells needs to be verified. LMP2 inhibition alone appears to be sufficient to suppress the secretion of inflammatory cytokines from microglial cells since inhibition of LMP2 but not Y was observed in Tg2576 mice treated with YU102. This result is highly intriguing given that inhibitors of LMP2, such as KZR-504, previously displayed little to no inhibition of inflammatory cytokine release in human PBMCs^39^. With this in mind, we suspect that LMP2 may have a cell type (or organ)-specific function in elevating cytokine generation. In microglial BV-2 cells, the potent LMP7-selective inhibitor ONX 0914 appears to be nearly as effective in suppressing inflammatory cytokine production as YU102. However, ONX 0914 was much more cytotoxic than YU102 in all tested cell lines. Overall, it suggests that LMP2/Y may offer a better therapeutic target than LMP7 in the development of therapeutic agents for neuroinflammatory diseases including AD.

Multiple previous studies have reported the detection of Aβ in RPE cells from age-related macular degeneration (AMD) patients as well as AD patients with early signs of AMD^75–79^. Based on these observations, it has been proposed that inflammation triggered by Aβ may represent a common pathological mechanism for AD and AMD^72,80–82^. In line with this, our results showed that treatment of YU102 not only improves cognitive deficits but also blocks RPE degeneration triggered by Aβ-induced inflammation in the APP transgenic mouse model. This potentially suggests that LMP2/Y could be a common therapeutic target for AD and AMD. In summary, we show here that YU102, a dual inhibitor of LMP2 and Y subunits, improves cognitive function in AD mouse models without affecting Aβ deposition and suppresses the secretion of inflammatory cytokines from microglial cells. This strongly indicates a link between LMP2/Y and microglia-mediated innate immune responses, potentially offering a common therapeutic strategy for treatment of neuroinflammatory disorders including AD.

## Materials and Methods

All experiments and methods were carried out in accordance with relevant guidelines and regulations. Specifically, all animal studies were approved by the Animal Care and Use Committee (IACUC) of Chungbuk National University (approval number: CBNUA-144-1001-01). All cell culture experiments were approved by the University of Kentucky Biosafety Committee (Approval No. B17-3000-M).

### Animals

For YU102 efficacy studies, 9-month-old Tg2576 and 8-week-old ICR mice were purchased from the Division of Laboratory Animal Resources (Korea FDA, Osong, South Korea) and Samtako (Osan, South Korea), respectively. Animals were housed three per cage, allowed access to water and food *ad libitum*, and maintained on a 12-h light/dark cycle regulated at 23 °C. Experiments were performed at least 1 week after their arrival in individual home cages.

### Tissue extraction from Tg2576 mouse

Tissues were extracted following a procedure previously reported^83^. The hippocampus was obtained from parasagittal brain slices; coronal brain slices were used to obtain nucleus accumbens (NAc) and striatum; the ventral tegmental area (VTA) was dissected from horizontal brain slices. Tissues were homogenized in RIPA buffer (containing 50 mM Tris-HCl (pH 7.5), 150 mM NaCl, 5 mM MgCl_2_, 1 mM EDTA, 1% Triton X-100, 0.25% sodium deoxycholate, 0.1% SDS, 1 mM sodium orthovanadate, 5 mM β-glycerophosphate, 5 mM NaF and protease inhibitor cocktail), sonicated and incubated on ice for 30 min. The samples were then centrifuged at 14,000 *g* (4 °C) for 20 min and the protein concentrations of the supernatant were determined by the Bradford method.

### Proteasome activity assay

Purified 20S human proteasomes (from Boston Biochem) were used to assess the *in vitro* activity of proteasome inhibitors. In 96-well format, 20 S proteasomes (0.5 μg/mL) were mixed with proteasome inhibitors in assay buffer (20 mM Tris-HCl, 0.5 mM EDTA, 0.035% SDS) at room temperature for 30 min, prior to the addition of fluorogenic substrates to a final assay volume of 100 μL. Fluorogenic substrates used in this study are: Suc-LLVY-AMC (CT-L activity, 100 μM), Ac-PAL-AMC (LMP2, 100 μM), Ac-WLA-AMC (β5, 20 μM), Ac-nLPnLD-AMC (β1,100 μM), and Ac-ANW-AMC (β5i, 100 μM). The fluorescence of liberated AMC was measured over a period of 90 min at excitation and emission wavelength of 360 and 460 nm, respectively, on a SpectraMax M5 fluorescence plate reader (Molecular Devices).

To measure proteasome activity in brain tissues isolated from Tg2576 mice, tissues were homogenized in RIPA buffer (50 mM Tris Cl, pH 7.4, 150 mM NaCl, 5 mM EDTA, 1% Nonidet P-40 substitute, 1% sodium deoxycholate, 0.1% SDS, 1% aprotinin, 50 mM NaF) and sonicated. Samples were then centrifuged for 20 min at 14,000 g (4 °C). Aliquots of supernatant containing an equivalent amount of total protein (by the Bradford protein assay) were added to a 96-well plate prior to the addition of fluorogenic substrate (Ac-PAL-AMC) at 37 °C. Fluorescence signals were recorded for 90 min using a Synergy-HT (Bio Tek) plate reader. Residual hydrolysis of the substrate (Ac-PAL-AMC) measured in lysates treated with 10 μM YU102 was regarded as non-proteasomal and subtracted from each experimental measurement of proteasome activity.

### Immunohistochemical staining

Immunohistochemical staining was performed as previously described^83^. Frozen hippocampal tissues were cut into 30 μm sections by using cryostat microtome (Leica CM1850, Leica Microsystems, Korea) For immunohistochemical staining, sections were rinsed in PBS and incubated in 3% hydrogen peroxide in PBS for 30 min. After washed in PBS for 10 min, the sections were incubated for 2 h at room temperature with rabbit polyclonal antibodies against Aß (1:300; Abcam, Inc, USA), Iba-1 (1:300, Wako, Japan) or a mouse polyclonal antibody against GFAP (1:300; Santa Cruz Biotechnology Inc., CA, USA). The sections were washed in PBS, followed by incubation with biotinylated goat anti-rabbit or anti-mouse IgG secondary antibodies (1:1000; GeneTex Inc., USA) for 1 hr at room temperature. in the presence of biotinylated goat anti-rabbit or anti-mouse IgG secondary antibodies. After washed and dehydrated, the sections were cleared in xylene and covered with Permount (n = 6 mice per group).

### Measurement of Aβ

Hippocampal Aβ_1–42_ levels were determined using an ELISA Kit (Cusabio Biotech Co., Ltd., Wilmington, DE, USA) according to the manufacturer’s instructions. In brief, samples and standards were pipetted into the pre-coated plate and incubated for 2 h at 37 °C. Biotinylated detection antibodies were added to each well and incubated 1 h at 37 °C. After washing, HRP-avidin was added and incubated for 30 minutes at 37 °C. After washing, Tetramethylbenzidine (TMB) substrate was added to each well. After the addition of stop solution, the absorbance was measured at 450 nm using a microplate reader (Sunrise, TECAN, Switzerland).

### Thioflavin T staining

Frozen hippocampal tissues were cut into 30 μm sections using a cryostat microtome (Leica CM1850; Leica Microsystems). The resulting pieces of tissue were thoroughly washed with distilled water for 5 min, and then transferred to gelatin-coated slides and placed in 1% Thioflavine T for 5 min, followed by ethanol dehydration (50%, 70%, 90%, and 100%, 2 min in each grade). The dehydrated samples were then mounted with mounting medium (Fluoromount, Sigma). Thioflavin T staining was examined by using a fluorescence microscope.

### Cresyl violet staining

Cresyl violet staining was performed as reported^83^. Frozen hippocampal tissues were cut into 30 μm sections by using cryostat microtome (Leica CM1850, Leica Microsystems, Korea). The pieces of tissues were fixed in 4% paraformaldehyde for 24 h at 4 °C. In order to identify cortical layers and cytoarchitectural features of the isocortical region, the post-fixed tissues were washed with PBS and then transferred to gelatin-coated slides and stained with 0.1% Cresyl violet (2–5 min). The sections were then washed with distilled water and dehydrated in 50%, 70%, 90%, and 100% ethanol for 2 min in each concentration. The tissues were airdried and immersed in a 1:1 mixture of absolute ethanol and xylene for 1 min. Following removal of the previous solution, the tissues were rinsed with xylene for 5–10 min and mounted with mounting medium (Cytoseal XYL, Thermo Scientific, USA). The matching areas of tissues were photographed at 100x magnification.

### Morris water maze test

Morris water maze test was performed following a procedure described previously^84^. Briefly, a circular plastic pool was filled with water and an escape platform was submerged 1–1.5 cm below the surface of the water. During the entire experiments, quiet environment and constant water temperature (22–25 °C) were maintained. Mice were trained three times a day with randomized starting points over 5 days. Each trial lasted for 60 s or ended immediately after the mice reached the submerged platform. Swimming was video-tracked, and the escape latency, escape distance and swimming speed were assessed by the SMART-LD program (Panlab, Spain). A probe test was performed 24 h after the water maze test (i.e. Day 6) to assess spatial memory consolidation. For the spatial probe test, the platform was removed from the pool and mice spent time searching the target area. The swimming pattern of each mouse for 60 s was recorded with a video-tracking system and analyzed by the SMART-LD program.

### Passive avoidance

Passive avoidance test was performed as previously described^84^. The passive avoidance test was given 48 h after the probe test. The passive avoidance response was determined using a “step-through” apparatus (Med Associates Inc., Vermont, USA) that is consisted of a lighted chamber and a dark chamber (each 20.3 × 15.9 × 21.3 cm) adjoining each other through a small gate. The grid floor was made of 3.175-mm stainless steel rods set 8 mm apart. For the learning trial, on the first day (i.e. Day 7), the mice were released in the illuminated compartment facing away from the dark compartment. As soon as mice moved into the dark compartment, mice received an electric shock (0.45 mA, 3 s duration). Testing trial was performed 24 h after learning trial (i.e. Day 8) and was procedurally identical to learning, except that electronic shock was not delivered. Each mouse was placed in the lighted compartment and the entrance latency until the mice re-entered the dark compartment was determined and defined as the step-through latency. Training was terminated when the mice in the light compartment for 120 consecutive seconds.

### Tau aggregation assay

HEK 293 tau-BiFC cells were maintained in DMEM containing 10% FBS, 100 units/mL penicillin, 100 μg/mL streptomycin, and 100 μg/mL G418 disulfate (Geneticin, Sigma) at 37 °C in a humidified atmosphere containing 5% CO_2_. For the treatment of IP selective inhibitors, HEK 293 tau-BiFC cells were plated on μ−clear 96-well plates. The next day, cells were incubated with YU102 or YU102 epimer co-treated with thapsigargin (1 μΜ) at various concentrations. After 46 h of incubation, the entire 96-well plate was imaged automatically by using Operetta (PerkinElmer, USA). The cellular intensities of tau-BiFC fluorescence were analyzed using Harmony 3.1 software. Error bars indicate S.D. from two independent experiments. Each experiment was performed as triplicate.

### Cell culture

Murine microglial cell line EOC-20, human myeloma cell line RPMI 8226, and human lung fibroblast cell line WI-38 were obtained from the ATCC (American Type Culture Collection) and cultured in DMEM-conditioned medium produced from the LADMAC cell line, RPMI 1640 medium, and EMEM medium respectively, according to manufacturer’s instructions. BV-2 murine microglial cell line was a kind gift of Dr. Won-Gon Kim (Korea Research Institute of Bioscience & Biotechnology, Korea) and cultured in DMEM medium. All cell lines were tested to have no mycoplasma contamination before experiments.

### Membrane-based cytokine array

A cytokine antibody array assay was performed with a mouse cytokine array kit (R&D Systems) according to the manufacturer’s protocol. Briefly, BV-2 cells, seeded in a 12-well plate at 2 × 10^5^ cells per well, were incubated with 1 μg/mL of *E. coli* 055:B5 lipopolysaccharide (Thermo Scientific) and 3 μM of YU102 or ONX 0914 for 24 h. The supernatants from BV-2 cells were collected and centrifuged to remove cell debris. The resulting supernatants were then incubated with assay membranes precoated with capture antibodies overnight at 4 °C. After rinsing the membranes with the manufacturer’s wash buffer, a detection antibody cocktail was added followed by the addition of streptavidin–horseradish peroxidase (HRP). In order to detect the immunoblot chemiluminescence on X-ray film (Thermo Scientific or GeneMate), SuperSignal West Femto Chemiluminescent Substrate (Thermo Scientific) was substituted for the manufacturer’s included substrate due to its higher signal strength and sensitivity.

### Cytokine enzyme-linked immunosorbent assay (ELISA)

BV-2 microglial cells (2.5 × 10^5^ cells/well) were seeded in 12-well culture plates. After overnight incubation, cells were simultaneously treated with 1 μg/mL of *E. coli* LPS and various concentrations of YU102, YU102 epimer, or ONX 0914 for 24 h. Supernatants were analyzed for the quantification of released pro-inflammatory cytokines, using Mouse IL-1α, IL-6, or CCL12/MCP-5 uncoated sandwich ELISA Kits (Thermo Scientific) on high-binding ELISA plates according to the manufacturer’s protocol. Briefly, standards and samples were incubated on capture antibody coated plate for 2 h at room temperature, followed by incubation with detection antibody for 1 h and then Avidin-HRP for 30 minutes. For visualization, tetramethylbenzidine substrate solution was added to each well, and then the reaction was stopped by the addition of stop solution (2N H_2_SO_4_). Absorbance was measured using a SpectraMax M5 microplate reader (Molecular Devices) at 450 nm wavelength. Serum IL-1α levels in Tg2576 mice were determined using a Quantikine ELISA kit (MLA00, R&D systems) according to manufacturer’s protocol. Mouse serum samples were diluted 5-fold prior to the ELISA. 50 µL of diluted samples or standards were incubated for 2 h at room temperature. After washing away any unbound substances, mouse polyclonal antibody specific to IL-1α was incubated for 2 h at room temperature. After washing 5 times, ELISA peroxidase substrate was added for visualization at 450 nm. The linear range of the ELISA system was between 4.69 and 300 pg/mL of IL-1α.

### Cell viability assay

BV-2, EOC-20, and WI-38 cells were seeded at 5,000 cells/well and RPMI 8226 cells were seeded at 10,000 cells/well in 96-well plates. Following overnight incubation, cells were treated with carfilzomib, ONX0914 or YU102 at indicated concentrations for 72 h. Cell viability was determined by CellTiter 96 AQueous One Solution Cell Proliferation assay (Promega) following the manufacturer’s protocol. Absorbance at 490 nm was measured using a SpectraMax M5 microplate reader (Molecular Devices).

### RPE flat mounts

Eyes of Tg2576 transgenic mice were fixed in 4% paraformaldehyde for 1 h at room temperature. Anterior eye cups were dissected in cold PBS. After removal of the retina, RPE sheets were permeabilized with 0.2% Triton X-100 for 15 min at room temperature. RPE sheets were then blocked with 3% BSA for 1 h at room temperature. Tissues were incubated with β-catenin (1:100; Abcam, ab19381) overnight at 4 °C. RPE sheets were rinsed in PBS containing 0.5% BSA and then incubated with Alexa 555-conjugated secondary antibody (1:1000; Invitrogen, A21422) for 2 h at room temperature and mounted. Samples were observed by using a confocal microscope (Carl Zeiss, LSM 800)

### Statistics

Results are expressed as means ± S.D. Statistical significance of the observed group differences was determined using Student’s t-test or two-way ANOVA followed by Dunnette’s *post hoc* or Bonferroni posttests test. Significance was set at p < 0.05 for all tests. All statistical analyses were carried out using GraphPad Prism 8.0.1 (GraphPad Software).

## Supplementary information

Supplementary information

## Data Availability

The data that support the findings of this study are available from the corresponding author upon reasonable request.
