# Supplementary material for: A dual inhibitor of the proteasome catalytic subunits LMP2 and Y attenuates disease progression in mouse models of Alzheimer’s disease
Source: Sci Rep. 2019 Dec 5;9:18393. doi: 10.1038/s41598-019-54846-z (PMC6895163; doi:10.1038/s41598-019-54846-z)

# Supplemental Information

# A dual inhibitor of the proteasome catalytic subunits LMP2 and Y attenuates disease progression in mouse models of Alzheimer's disease

In Jun Yeo,[1,#] Min Jae Lee,[2,#] Ahruem Baek,[3] Zachary Miller,[2] Deepak Bhattarai,[2] Yu Mi Baek,[3] Hyun Jung Jeong,[4] Yun Kyung Kim,[4] Dong-Eun Kim,[3] Jin Tae Hong[1,*] and Kyung Bo Kim,[2,*]

[1]College of Pharmacy, Chungbuk National University, Cheongju, Chungbuk 28160, Republic of Korea; [2]Department of Pharmaceutical Sciences, University of Kentucky, Lexington, KY 40536-0596, USA; [3]Department of Bioscience and Biotechnology, Konkuk University, Seoul 05029, Republic of Korea; [4]Korea Institute of Science and Technology (KIST), Brain Science Institute, Convergence Research Center for Diagnosis, Treatment and Care System of Dementia, Seoul 02792, Republic of Korea

**Contents:**

**S-1**: Title Page

**S-2**: Supplemental Figure 1. Initial screening of PR-825, PR-924 (IPSI) in LPS-induced mouse model of neuroinflammation.

**S-2**: Supplemental Figure 2. Remaining proteasome activities (CT-L and LMP2) in heart and lung tissues collected from Tg2576 mice treated with YU102 or YU102 epimer.

**S-3**: Supplemental Figure 3. Interaction between YU102 and ABCB1 using ABCB1-overexpressing cell line (RPMI 8226/ABCB1).

**S-4**: Supplemental Figure 4. Full immunohistochemistry results for GFAP, Iba-1, and COX-2 in the hippocampal tissues of Tg2576 mice treated with vehicle, YU102, or YU102 epimer.

**S-5:** Supplemental Figure 5. Full suppression profile of cytokine production by YU102 in LPS-stimulated BV-2 cells.

**S-7:** Supplemental Figure 6. Cell viability graphs for YU102, and ONX 0914 in various cell lines.

**Supplemental Figure 1.** Spatial recognition memory was evaluated by the Morris water maze test: escape latency time in the target quadrant (left) and escape distance of the mice (right). 8-week old ICR mice were treated with daily injections of LPS for 5 days, followed by treatment with YU102 (10 mg/kg), PR-924 (10 mg/kg), and PR-825 (2 mg/kg) twice a week for 3 weeks. At the end of the treatment period, the Morris water maze test was performed.

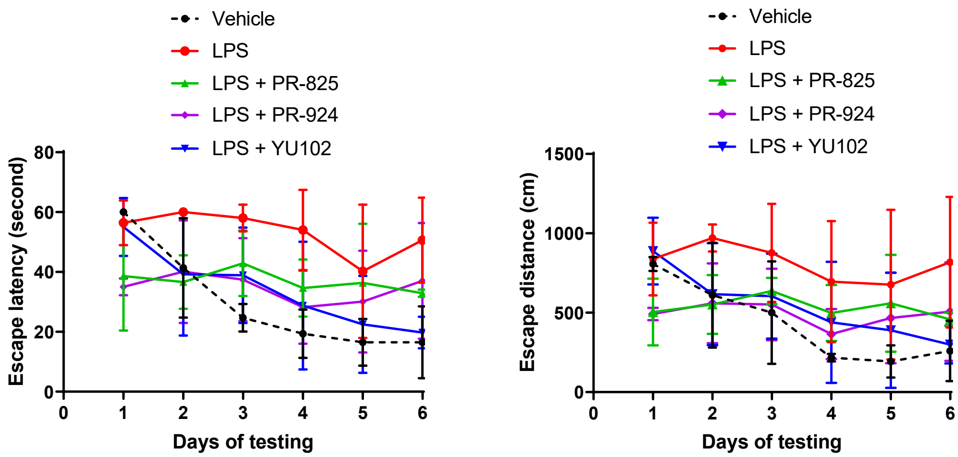


**Supplemental Figure 2.** Upon the completion of the behavior test, proteasome activities in heart and lung collected from Tg2576 mice treated with vehicle, YU102 (10 mg/kg), or YU102 epimer (10 mg/kg) were measured using fluorogenic substrates. Error bars are standard deviation derived from three technical replicates. *Differences in LMP2 inhibitory activity in heart and lung tissues between vehicle-treated and YU102-treated group or YU102-treated and YU102 epi-treated group were statistically significant (p-value < 0.05, n=3).

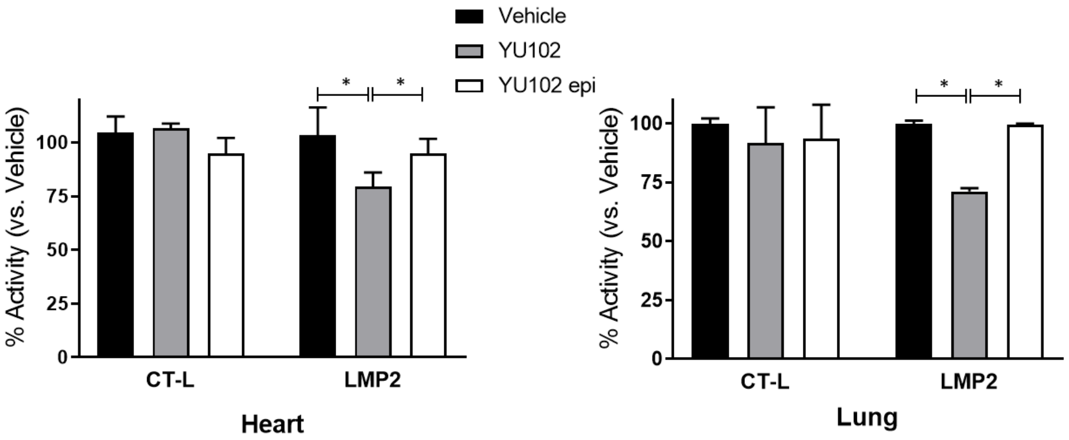

**Supplemental Figure 3.** Interaction between YU102 and ABCB1 using ABCB1-overexpressing cell line (RPMI 8226/ABCB1). Cells pretreated with/without reversin 121 (7.5μM) were incubated with compounds (1 μM) for 4 hr, followed cell lysis and proteasome kinetics. Proteasome assay was performed using LMP2-specific substrate. CT-L activity was measured since Cfz known as a ABCB1 substrate is a CT-L inhibitor. *Differences in remaining proteasome activity after incubating compounds between vehicle-pretreated and reversin 121 pretreated were statistically significant.

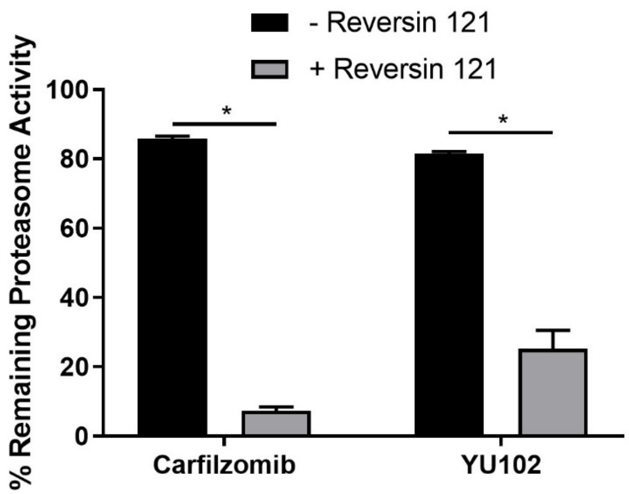

**Supplemental Figure 4.** Immunohistochemical stainings were performed for GFAP (a), Iba-1 (b), and COX-2 (c) using frozen hippocampal tissues of vehicle-treated, YU102-treated, or YU102 epimer-treated Tg2576 mice (n=3 mice per group).

**a. GFAP**

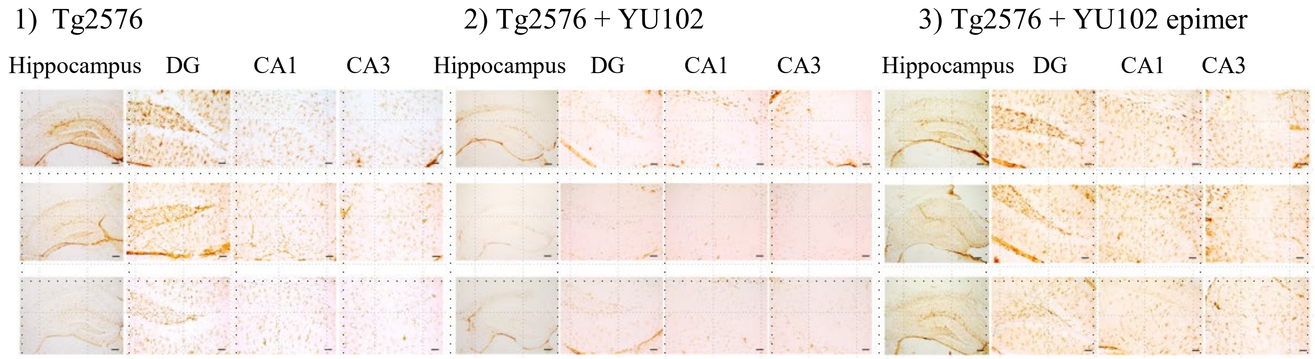


**b. Iba-1**

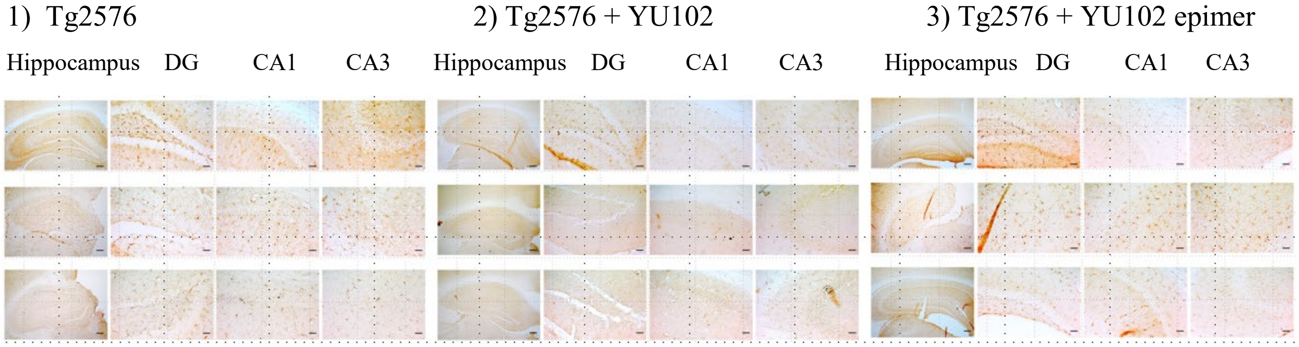


**c. COX-2**

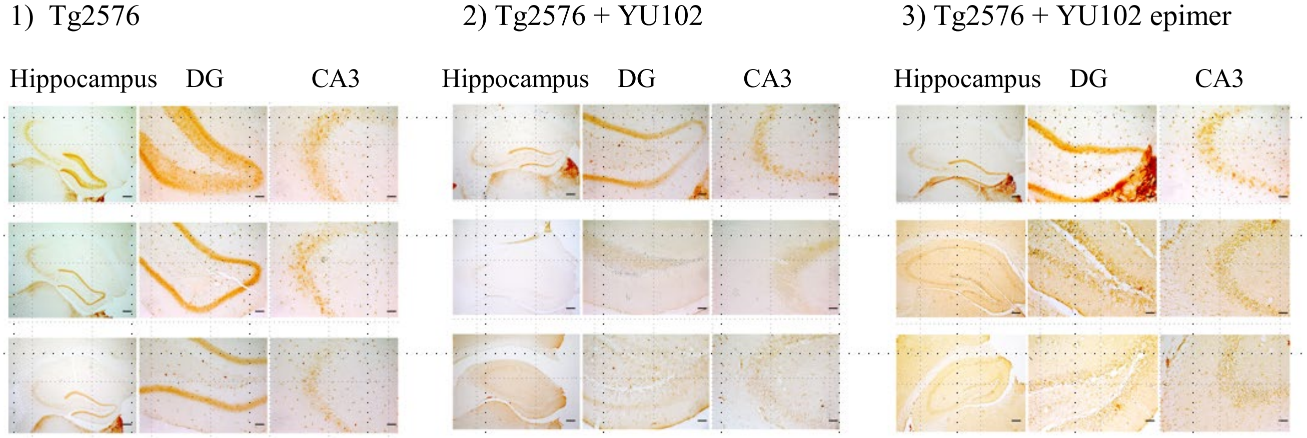

**Supplemental Figure 5.** Full suppression profile of cytokine production by YU102 in LPS-stimulated BV-2 cells using a mouse cytokine array kit (R&D Systems). BV-2 cells were treated with vehicle, LPS (1μg/mL) alone, and YU102 (3μM) or ONX0914 (3μM) with LPS (1μg/ mL). Four membranes were exposed to an X-ray film for 1, 3, 5, or 10 minutes (a). The amount of each cytokine or chemokine was relative to the mean of the intensity of corresponding spots from vehicle control sample**.** Each cytokine or chemokine has duplicate detection spots. Graph depicts the mean spot pixel density from the arrays using Quantity One software (Bio-rad) analysis (b).

**a.**

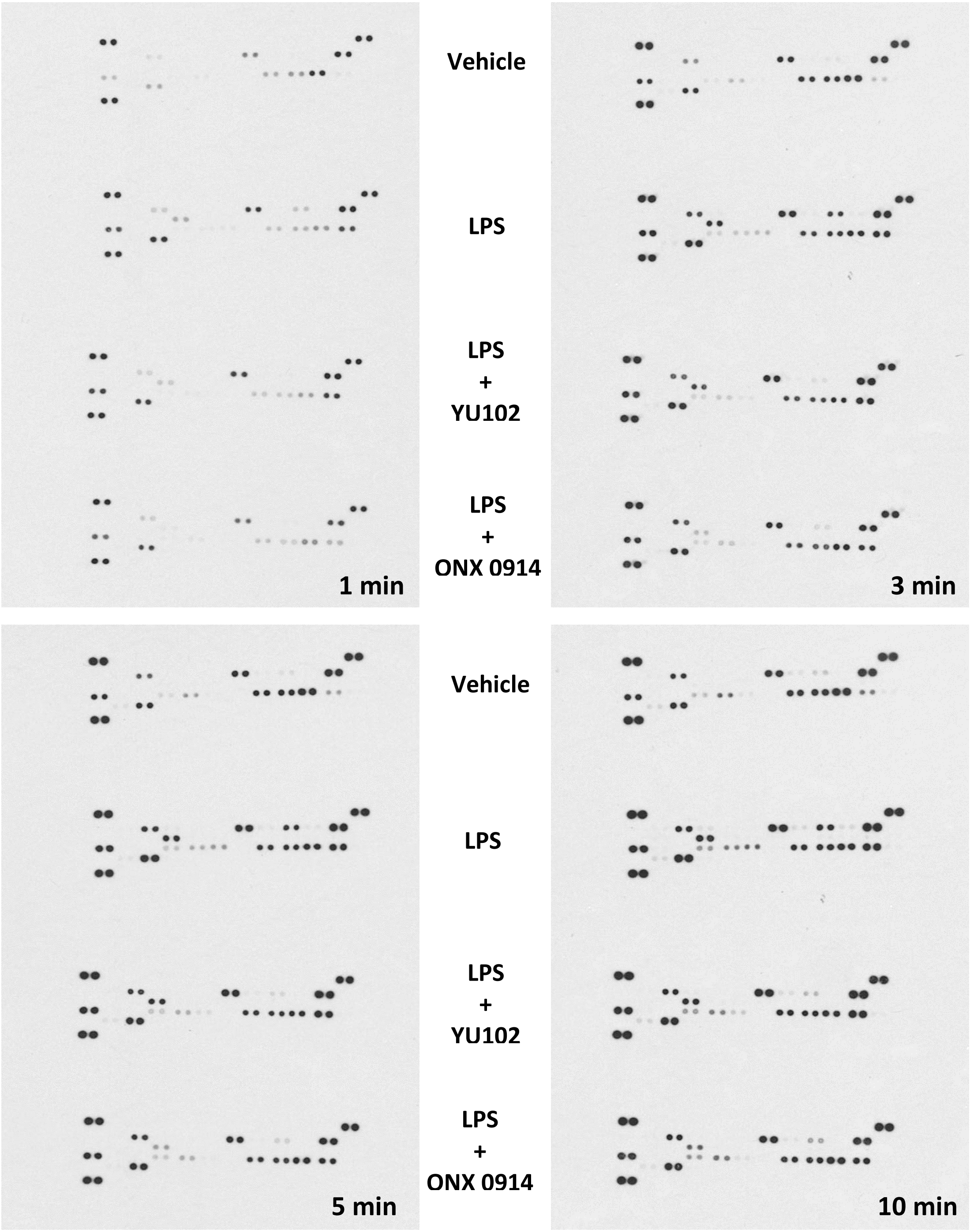

**b.**

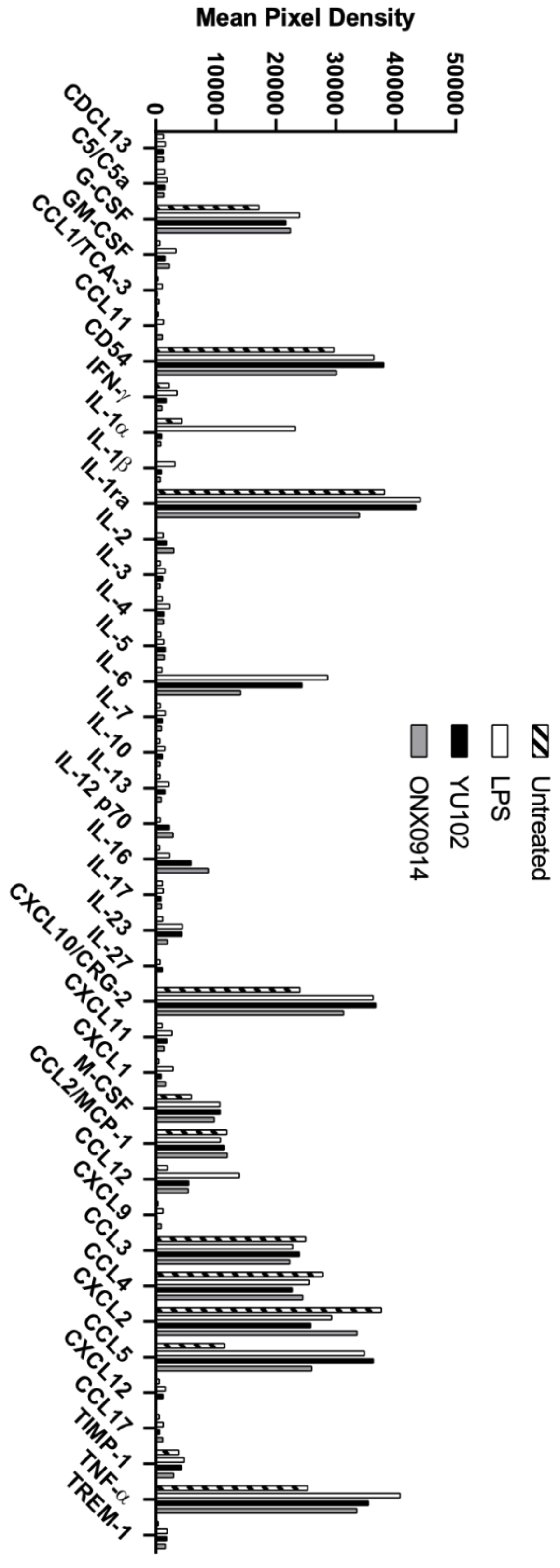
Mean Pixel Density
0
10000
20000
30000
40000
50000
CDCL13
C5/C5a
G-CSF
GM-CSF
CCL1/TCA-3
CCL11
CD54
IFN-γ
IL-1α
IL-1β
IL-1ra
IL-2
IL-3
IL-4
IL-5
IL-6
IL-7
IL-10
IL-13
IL-12 p70
IL-16
IL-17
IL-23
IL-27
CXCL10/CRG-2
CXCL11
CXCL1
M-CSF
CCL2/MCP-1
CCL12
CXCL9
CCL3
CCL4
CXCL2
CCL5
CXCL12
CCL17
TIMP-1
TNF-α
TREM-1
Untreated
LPS
YU102
ONX0914

**Supplemental Figure 6**. Cell viability graphs for YU102 and ONX0914 in various cell lines. EOC-20 and WI-38 cells were seeded at 5,000 cells/well and RPMI 8226 cells were seeded at 10,000 cells/well in 96-well plates. Following overnight incubation, cells were treated with YU102 or ONX0914 at indicated concentrations for 48 h (EOC20 with or without 24h 1μg/mL LPS pretreatment) or 72 h (RPMI 8266 and WI-38). Cell viability was determined by CellTiter 96 AQueous One Solution Cell Proliferation assay (Promega) following manufacturer's protocol. Absorbance at 490 nm was measured using a SpectraMax M5 microplate reader (Molecular Devices).

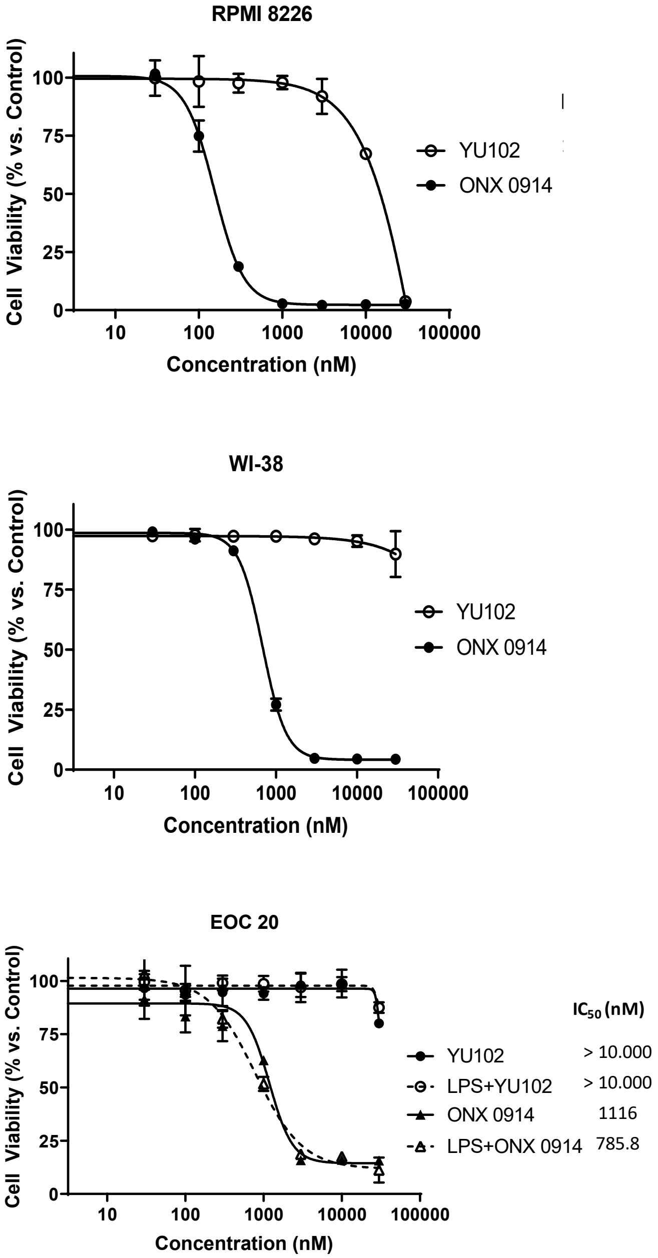

Supplement: Supplementary file 1 — Supplementary information [file 41598_2019_54846_MOESM1_ESM.pdf]
